# Further characterization of *Maize chlorotic mottle virus* and its synergistic interaction with *Sugarcane mosaic virus* in maize

**DOI:** 10.1038/srep39960

**Published:** 2017-01-06

**Authors:** Qiang Wang, Chao Zhang, Chunyan Wang, Yajuan Qian, Zhenghe Li, Jian Hong, Xueping Zhou

**Affiliations:** 1 State Key Laboratory for Biology of Plant Diseases and Insect Pests, Institute of Plant Protection, Chinese Academy of Agricultural Sciences, Beijing, 100193, People’s Republic of China; 2 State Key Laboratory of Rice Biology, Institute of Biotechnology, Zhejiang University, Hangzhou 310029, People’s Republic of China

## Abstract

Maize chlorotic mottle virus (MCMV) was first reported in maize in China in 2009. In this study we further analyzed the epidemiology of MCMV and corn lethal necrosis disease (CLND) in China. We determined that CLND observed in China was caused by co-infection of MCMV and sugarcane mosaic virus (SCMV). Phylogenetic analysis using four full-length MCMV cDNA sequences obtained in this study and the available MCMV sequences retrieved from GenBank indicated that Chinese MCMV isolates were derived from the same source. To screen for maize germplasm resistance against MCMV infection, we constructed an infectious clone of MCMV isolate YN2 (pMCMV) and developed an *Agrobacterium*-mediated injection procedure to allow high throughput inoculations of maize with the MCMV infectious clone. Electron microscopy showed that chloroplast photosynthesis in leaves was significantly impeded by the co-infection of MCMV and SCMV. Mitochondria in the MCMV and SCMV co-infected cells were more severely damaged than in MCMV-infected cells. The results of this study provide further insight into the epidemiology of MCMV in China and shed new light on physiological and cytopathological changes related to CLND in maize.

Maize chlorotic mottle virus (MCMV) is the only member of the genus *Machlomovirus* in the family *Tombusviridae*. The 4436-nucleotide, sense, single-stranded RNA of the MCMV genome is encapsidated in isometric particles about 30 nm in diameter. Six open reading frames (ORFs) have been reported for the MCMV genome^1^,^2^. ORF1 encodes a 32-kDa hypothetic protein of unknown function. ORF2 encodes a 50-kDa protein (P50) and an N-terminus-overlapped 111 kDa protein (P111) produced by translational read-through of the UAG stop codon of ORF2. ORF4 encodes a 7-kDa protein, which functions as the movement protein of MCMV. A 31-kDa protein is expressed from ORF5 when the UGA stop codon of ORF4 is suppressed; it also has a role in cell-to-cell movement of MCMV^3^. The coat protein (CP) of MCMV is expressed from the 3′ proximal ORF (Fig. 1). When maize plants are co-infected with MCMV and one of several potyviruses including maize dwarf mosaic virus (MDMV), wheat streak mosaic virus (WSMV) or sugarcane mosaic virus (SCMV), leaves and stems of infected plants develop a severe systemic necrosis known as corn lethal necrosis disease (CLND). CLND is an important disease in maize industry in many countries^4^.

MCMV was first reported in Peru and later in the United States^5^,^6^,^7^. MCMV is transmitted by mechanical inoculation and seed, and it was also reported to be transmitted by chrysomelid beetles and thrips^8^. In 2009, MCMV was first observed in China in maize with necrotic and chlorotic leaves and stems^9^. At that time, an unknown virus with flexuous rod-shaped particles was also observed in the cytoplasm of infected leaf cells using electron microscopy. In the present study, we surveyed the distribution of MCMV and CLND in ten provinces of China by ELISA and RT-PCR. The results show that MCMV currently occurs only in the Yunnan and Sichuan Provinces of China, and the CLND disease observed in China is caused by co-infection with MCMV and SCMV. An infectious clone of MCMV was constructed by inserting the full length cDNA of representative MCMV isolate YN2 (MCMV-YN2) downstream a duplicated cauliflower mosaic virus (CaMV) 35S promoter, and an *Agrobacterium*-mediated injection procedure was developed to allow high throughput inoculation of maize with the MCMV infectious clone. We also compared the ultrastructural changes in cells infected with MCMV alone to those co-infected with MCMV and SCMV.

## Results

### Occurrence of MCMV and CLND in China

To determine which potyvirus was present in the CLND maize plant reported by Xie *et al*.^9^, we analyzed that sample using RT-PCR with a set of universal primers; a 1.8-kb product was amplified and then cloned and sequenced. The result showed that this fragment shared 99% sequence identity with the SCMV Vietnam isolate (DQ925426) and the CP coding sequence within this fragment showed 99% sequence identity with the reported CP sequence of SCMV Thailand isolate (AM501533) (data not shown). The ELISA using specific antibodies also showed the presence of SCMV but not MDMV or WSMV. To screen for the presence of MCMV and SCMV in the 171 maize samples collected from 10 maize-growing provinces in China from 2009 to 2013, samples were tested with ELISA using MCMV or SCMV specific monoclonal antibodies and ELISA kits specific for MDMV or WSMV. The results showed that 20 samples were infected by SCMV, and 33 samples were co-infected by MCMV and SCMV (Table 1). No sample was infected by MDMV or WSMV. Interestingly, none of the samples analyzed in this study were infected by MCMV alone, and all samples co-infected with MCMV and SCMV were from Yunnan and Sichuan Provinces. The MCMV and SCMV infections were confirmed through RT-PCR using specific primers (data not shown).

### Phylogenetic relationship among MCMV isolates

Four full-length genomic cDNAs representing four respective MCMV isolates were cloned and sequenced. These full-length sequences have been deposited in the GenBank database (GU138674 for isolate YN1; JQ982468 for isolate YN2; JQ982469 for isolate YN3 and JQ982470 for isolate Sch1). The phylogenetic relationship among these four isolates, a sugarcane MCMV isolate found in Yunnan^10^ (KF010583), a Taiwan MCMV isolate^11^ (KJ782300) and two American MCMV isolates^1^,^12^ (X14736 and EU358605) were analyzed. The results indicated that the isolates from Chinese mainland and Taiwan clustered in one group and the two isolates from America in the second group (Fig. 2), even though the sequence identity between the two groups is high (96.9–97.3%). Because the five isolates in group I shared 99.1%–99.7% sequence identity with each other, it is likely that all these isolates came from the same ancestor virus strain.

### *Agrobacterium*-mediated injection of MCMV in maize

**Figure 1:**
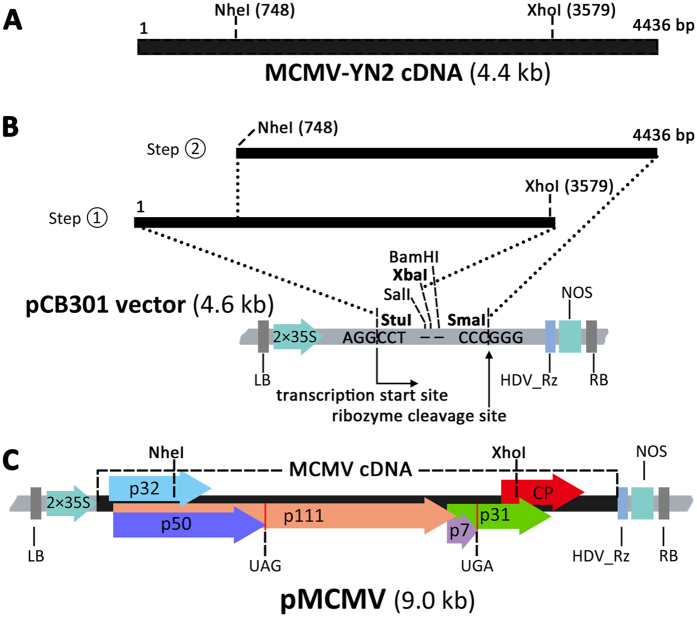
Schematic diagram of the construction strategy for pMCMV. (**A**) Full-length MCMV genome showing positions of restriction sites. (**B**) PCR-amplifed digestion products that were sequentially inserted into pCB301 to produce pMCMV. Positions of duplicated 2 × 35S promoter, hepatitis delta virus antigenomic ribozyme (HDV_Rz) and NOS terminator in pCB301 are indicated. Transcriptional start site and the HDV_Rz cleavage site are indicated. (**C**) Organization of MCMV cDNA between the StuI and SmaI sites. Positions of individual MCMV ORFs are shown. The read-through stop codons for p50 or p7 are also indicated.

**Figure 2:**
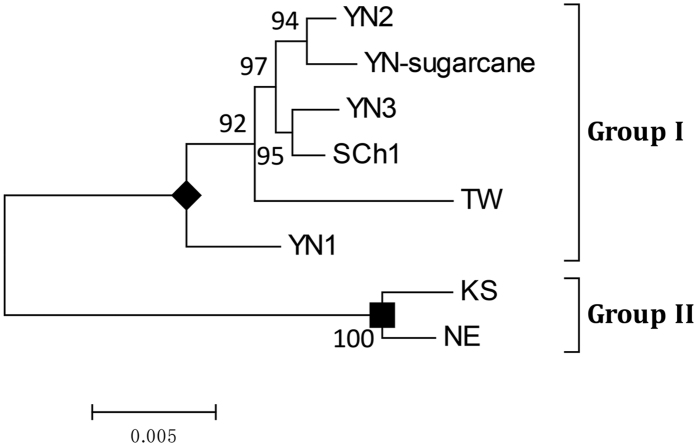
Phylogenic tree of MCMV isolates. The phylogenic tree was constructed with the neighbor-joining method in Mega5 with 1,000 bootstrap replicates. Scale bar indicates nucleotide substitutions per site. YN, Yunnan isolate; SCh, Sichuan isolate; TW, Taiwan isolate; KS, Kansas isolate; NE, Nebraska isolate.

**Figure 3:**
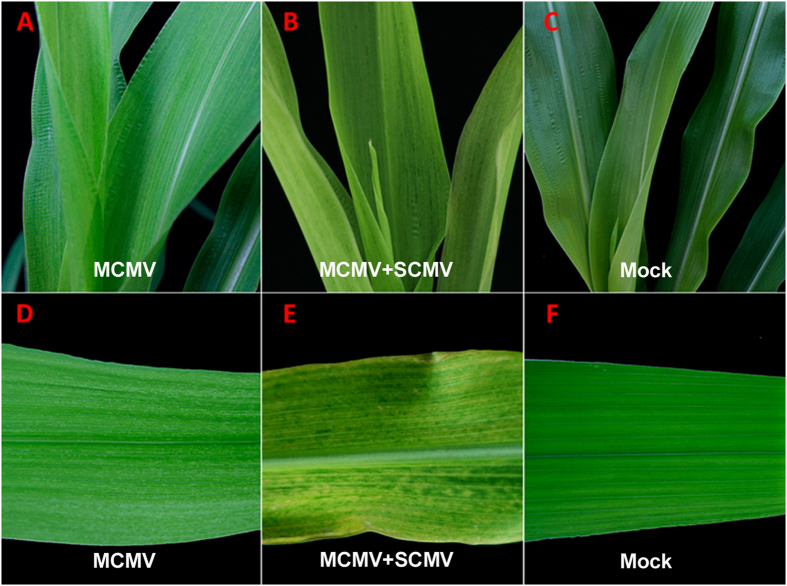
Symptoms on maize plants 15 d after agroinjection with pMCMV or co-infected with MCMV and SCMV. (**A**,**D**) Light chlorotic mottling on plants agroinjected with pMCMV. (**B**,**E**) Symptoms of MCMV and SCMV co-infection. (**C**,**F**) Negative controls agro-injected vector pCB301 (Mock).

The MCMV infectious clone pMCMV was constructed (Fig. 1). To establish an efficient inoculation procedure for maize, we transformed *Agrobacterium tumefaciens* GV3101 with the pMCMV plasmid and the transformed *Agrobacterium* cells were injected into stems of maize cv. B73 seedlings. Fifteen among the 29 inoculated maize plants developed light chlorotic mottling on newly developed leaves at 10 days post inoculation (dpi) (Fig. 3A,D), the symptoms were similar to that observed on MCMV-YN1-inoculated plants (data not shown) at 6 dpi, but were very distinct from that of MCMV and SCMV co-infected plants (Fig. 3B,E). All of the leaf samples showing mottling symptoms were infected with MCMV when analyzed by RT-PCR using primers MCMV/CP-F and MCMV/CP-R. No mottling symptoms were observed on the leaves of plants inoculated with *Agrobacterium* harboring the pCB301 empty vector (Fig. 3C,F). Inoculation experiments were repeated three times, and infectivity of pMCMV on B73 is given in Table 2.

**Figure 4:**
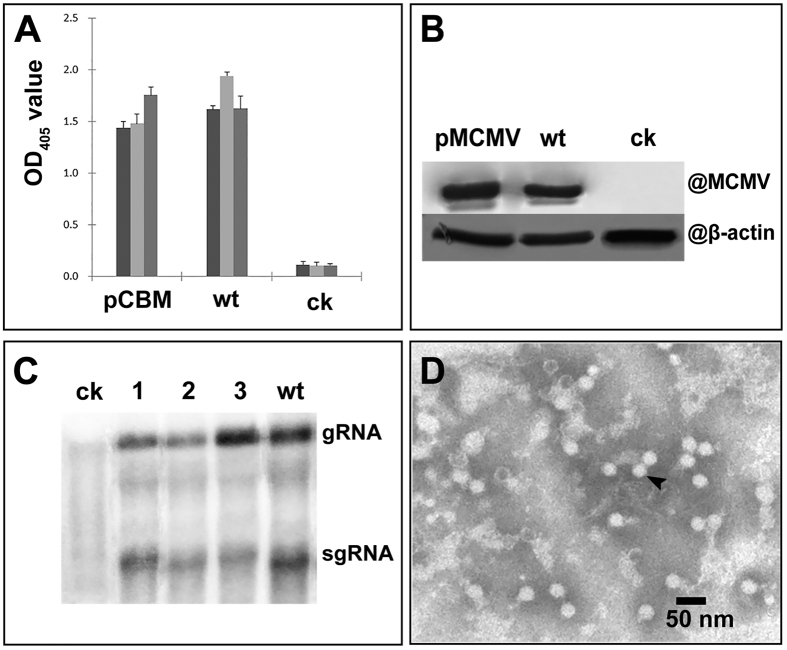
Detection of MCMV in pMCMV-inoculated maize plants. (**A**) Relative accumulation of MCMV in three plants detected by ELISA and absorbance at OD_405_. Three plants were tested for each treatment, and each sample was tested in triplicate. (**B**) Western blot of MCMV using MCMV-specific monoclonal antibody. Membrane probed with a monoclonal antibody against β-actin was used as loading control. (**C**) Northern blot assay for MCMV RNA using a probe was specific for MCMV CP region amplified by PCR with primers MCMV/CP-F and MCMV/CP-R. Lanes 1–3, three respective pMCMV-inoculated plants. (**D**) TEM of negatively stained viral particles (arrow) from pMCMV inoculated plants. ck, mock-inoculated plants; pMCMV, plants inoculated with pMCMV; wt, plants rub-inoculated MCMV-YN1. Leaves were harvested at 10 dpi in (**A**,**B**,**C** or **D**).

To determine the levels of MCMV in the infected maize plants, crude leaf extracts were isolated from the pMCMV-inoculated or MCMV-YN1-inoculated plants and analyzed by ELISA using MCMV antibody. Results showed that at 10 dpi the virus levels in the pMCMV-inoculated and MCMV-YN1-inoculated (wt) plants were similar (Fig. 4A). Western blot analysis of leaf extracts harvested at 10 dpi gave similar results for the pMCMV-inoculated or MCMV-YN1-inoculated plants (Fig. 4B). The Northern blots indicated that the relative level of viral RNA level in plants inoculated with pMCMV was comparable to that in plants inoculated with MCMV-YN1 (Fig. 4C). Numerous isometric particles of about 30 nm in diameter were also observed in negatively stained leaf extracts when checked with transmission electron microscope (Fig. 4D).

To investigate the infectivity of MCMV in different maize cultivars, we agro-inoculated maize cvs. B73, Zhengdan958, Suyu No.1, Huatian-Waxy Corn 072, Urban Beauty, Nongda108, Zhe-Waxy Corn no.5 with pMCMV. Virus infection in these cultivars were 72.2%, 50.0%, 58.3%, 83.3%, 77.8%, 77.8% and 97.2%, respectively, indicating that different maize cultivars have different tolerance reaction.

### Ultrastructural changes in infected maize leaf cells

**Figure 5:**
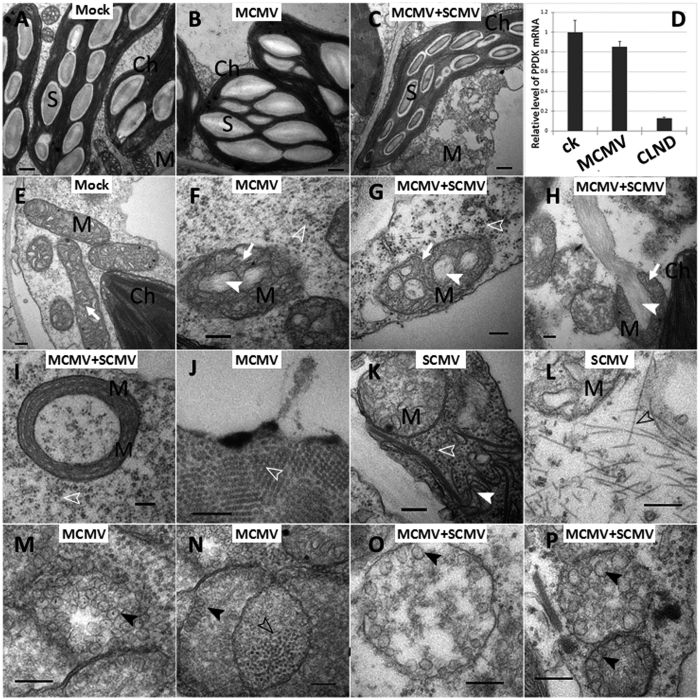
Transmission electron micrographs of maize cells and mRNA levels for PPDK in plants infected with MCMV alone, with MCMV and SCMV, or mock-inoculated with phosphate buffer. (**A**–**C**) Starch grains in chloroplasts in the bundle sheath cells of the mock-inoculated (**A**), MCMV-inoculated (**B**) or dual inoculation with MCMV and SCMV (**C**). (**D**) Quantitative RT-PCR to show relative PPDK expression. (**E**–**I**) Mitochondria in the mock- (**E**), MCMV- (**F**) and MCMV + SCMV-inoculated (**G**,**H**,**I**) cells. Disorganized cristae are marked with white arrows, and fine fibrous materials are marked with white solid arrowheads. White solid arrow in (**H**) indicates materials released from a disrupted mitochondria. (**J**) Aggregates of MCMV virions (white open arrowheads) in MCMV-infected plant cells. (**K**) Pinwheels (white solid arrowheads) in the SCMV infected cells. (**L**) Flexuous, rod-shaped virions (black open arrowheads) in the SCMV infected cells. Multi-vesicular bodies (black solid arrows) in MCMV-infected (**M**,**N**) or MCMV and SCMV co-infected cells (**O**,**P**). Spherical viral particles (black open arrowheads) were seen inside the Multi-vesicular bodies in (**N**). Ch, chloroplast; M, mitochondria; S, starch grains. Bars: a–c = 500 nm, e–p = 200 nm.

Because MCMV and SCMV co-infection is common in maize fields in Yunnan and Sichuan Provinces, we investigated the ultrastructural damage caused by MCMV infection and by MCMV and SCMV co-infection. Chloroplasts in bundle sheath cells of maize leaves are known to contain large starch grains and unstacked thylakoid membranes^13^. In the present study, bundle sheath cells infected with MCMV alone had starch grains in chloroplasts similar to those observed in the mock-inoculated plants (Fig. 5A and B), but cells co-infected with MCMV and SCMV, however, had much smaller starch grains in the chloroplasts (Fig. 5C). Results of qRT-PCR agreed with these ultrastructural observations and showed that the mRNA level of *pyruvate orthophosphate dikinase* (PPDK) gene, a rate-limiting factor for CO_2_ fixation in the C_4_-photosynthesis pathway^14^, was 7-fold lower in the co-infected leaf tissues than in the mock-inoculated or MCMV-infected plants (Fig. 5D).

Mitochondria in the MCMV-infected (Fig. 5F) or MCMV and SCMV co-infected cells (Fig. 5G to I) had disorganized cristae (white arrows) and fine, fibrous materials (white solid arrowheads) comparable to the case in mock-inoculated plant cells (Fig. 5E). In MCMV and SCMV co-infected cells, some mitochondria were heavily disrupted, leading to leakages of internal content (Fig. 5H, white solid arrow). Spherical MCMV virions were dispersed in the cytoplasm of the MCMV-infected cells (Fig. 5F and J, white open arrowheads) and in MCMV and SCMV co-infected cells (Fig. 5G to K, white open arrowheads). Typical pinwheel-like inclusions (Fig. 5K, white solid arrowheads) and flexuous filamentous virus particles (Fig. 5L, black open arrowheads) of SCMV were observed only in the co-infected cells. Multi-vesicular bodies (MVBs, black solid arrows) were found in both MCMV-infected cells (Fig. 5M and N, black solid arrows) and in cells co-infected with MCMV and SCMV (Fig. 5O and P, black solid arrows). Some spherical viral particles were observed within or between MVBs (Fig. 5N, black open arrowheads), suggesting they are potential sites for viral replication.

## Discussion

MCMV was first reported in Peru in 1974 and later in America and Thailand^1^,^3^,^5^,^6^,^15^,^16^,^17^. In the first report of MCMV in China in 2011^9^, an unknown flexious rod-shaped virus was observed in tissue samples by electron microscopy. Because MCMV was recently reported in several East Africa countries and Asia, and CLND has caused significant yield losses to maize production^18^,^19^,^20^,^21^, we decided to further investigate the occurrence of MCMV and CLND in China and to identify the unknown flexuous, rod-shaped virus that was observed in MCMV-infected maize samples^9^. The results of our field survey, started in 2009, of MCMV and CLND in maize-growing provinces in China, showed that MCMV currently occurs only in Yunnan and Sichuan Provinces in China. Phylogenic analysis indicated that the Chinese MCMV isolates were distinct from the reported American MCMV isolates.

Studies on the interactions between a virus and its host plant require an easy and effective inoculation procedure. Scheets and co-workers^22^ reported previously that *in vitro* transcribed MCMV RNA could be used to infect maize protoplasts and plants. Although this inoculation procedure is effective, it is costly and labor intensive. To develop an easy, low cost inoculation protocol for MCMV, we constructed a DNA-based MCMV vector and adopted an *Agrobacterium*-mediated injection procedure for maize. Because a large quantity of *Agrobacterium* can be cultured overnight at a very low cost, this protocol should benefit researchers doing large-scale screenings for maize genotypes resistant to MCMV infection.

Scheets *et al*.^22^ reported previously that MCMV RNA transcripts transcribed by an *in vitro* T7 promoter had an extra 5′ m^7^GpppG and were less infectious in maize protoplasts and did not infect maize plants. The transcription site of T7 polymerase might start at the + 2 position on the MCMV cDNA. Consequently, the 5′ end of the first adenylate residue was eliminated during the *in vitro* transcription^22^. The pMCMV vector was constructed by inserting the full-length MCMV cDNA between a duplicated CaMV 35S promoter and an HDV-ribozyme sequence. Transcription of MCMV genomic RNA through this 35S promoter ensures transcription from the 5′ end of MCMV, while the HDV-ribozyme cuts the 3′ end sequence of MCMV RNA to give an authentic 3′ end (Fig. 1)^23^,^24^. The MCMV RNA transcripts produced *in vivo* by the 35S promoter can infect maize plants as efficiently as the MCMV virus does.

A previous study showed that CLND-infected cells contain vacuolated MCMV viroplasms and pinwheel structures of SCMV^25^. In this study, chloroplasts in cells co-infected with MCMV and SCMV contained much smaller starch grains than that in the MCMV-infected cells, suggesting that photosynthesis in these cells was significantly impeded. We analyzed PPDK expression in MCMV or MCMV and SCMV co-infected cells through quantitative RT-PCR and the expression of this gene in leaves co-infected with MCMV and SCMV was indeed decreased. Mitochondria generate usable energy through the Krebs or tricarboxylic acid (TCA) cycle during plant growth and development. We observed that mitochondria in the co-infected leaf cells were severely damaged much earlier in the infection than in MCMV-infected cells. We consider that earlier disruption of chloroplast photosynthesis and mitochondrial respiration in the co-infected plants might be the main cause of the systemic necrosis in the CLND plants.

Interestingly, Multi-vesicular bodies (MVBs) were observed in both singly infected and co-infected plants. The MVBs found in MCMV-infected plants were morphologically similar to the peroxisomal multi-vesicular bodies (pMVBs) induced in *N. benthamiana* cells by tomato bushy stunt virus (TBSV, type species of the genus *Tombusvirus* in the family *Tombusviridae*)^26^. The pMVBs are the site of TBSV replication, we thus speculated that the MVBs are the scaffold for MCMV replication.

The concentration of MCMV increases over 5 fold in plants that are co-infected with SCMV, but the concentration of SCMV in co-infected plants is no difference than in singly infected plants^15^. Similar synergistic interactions were also observed in plants infected with potato virus X (PVX) and potato virus Y (PVY) or tobacco etch virus (TEV)^27^,^28^. Double infection of tobacco plants with PVX and PVY, PVX and TEV, or PVX with another potyvirus all induced much more severe symptoms and increased the level of PVX RNA. Numerous studies have also shown that potyviral HC-Pro, an RNA silencing suppressor, plays a critical role in the synergism during co-infection with PVX and a potyvirus^29^,^30^. Further study using transgenic tobacco expressing the TEV P1/HC-Pro showed a synergistic response upon infection of the plant with PVX. In contrast, tobacco plants expressing a mutant HC-Pro that could not suppress RNA silencing also failed to enhance PVX infection in the co-infected plants^29^. HC-Pro of SCMV is also a suppressor of RNA silencing^31^. This finding may explain why MCMV alone causes only mild disease symptoms in the infected plant, and the systemic necrosis and the increased MCMV RNA accumulation that result from the synergism between MCMV and SCMV in co-infected plants may be due to the presence of SCMV HC-Pro protein.

## Materials and Methods

### Virus sources

A Yunnan isolate of MCMV was previously identified in maize in Yunnan Province, China^9^ and maintained in maize cv. B73 in an insect-proof greenhouse. The maize plant was co-infected with MCMV and one potyvirus and developed lethal necrosis. Because MCMV can tolerate temperatures up to 85 °C for 10 min but potyviruses can tolerate only about 50–60 °C, leaf samples with CLND symptoms were treated for 10 min at 70 °C before inoculation of maize seedlings. Single infection with MCMV was thus obtained, and the virus isolate was named MCMV-YN1 and used as a control for MCMV inoculation.

To determine the distribution of viruses in maize in China, we collected 171 maize leaf samples with virus-like symptoms between 2009 and 2013 from 10 maize-growing provinces (Yunnan, Sichuan, Guizhou, Guangxi, Shandong, Liaoning, Heilongjiang, Henan, Hebei and Zhejiang).

### ELISA, RT-PCR and cloning

Monoclonal antibodies against MCMV^32^ and SCMV^33^, previously produced in our laboratory, were used to analyze leaf samples for the presence of MCMV and SCMV according to the procedure described previously^32^. MDMV and WSMV were identified using ELISA kits specific for MDMV or WSMV from Agdia (Elkhart, IN, USA) as instructed by the manufacturer.

Three MCMV samples (YN1, YN2 and YN3) from Yunnan province and one (Sch1) from Sichuan province showing lethal necrosis symptom were selected randomly for full-length genomic cDNA cloning and sequencing. RNA for RT-PCR was extracted from approximately 100 mg tissue from each leaf sample using TRIzol reagent (Invitrogen, Carlsbad, CA, USA). Two microliters of total RNA solution was then used in each 20-μL reverse transcription reaction using AMV Reverse Transcriptase XL (TAKARA, Kyoto, Japan) and the specific primer M2R. MCMV fragments were amplified by PCR using the KOD FX DNA polymerase (TOYOBO, Osaka, Japan). Primers M1F and M2R were designed based on the MCMV genome sequences available in the GenBank database (accessions GU138674, X14736 and EU358605) and used to amplify the full length MCMV cDNAs. The resulting cDNAs were inserted into separate pGEM-T Easy Vector (Promega, Madison, WI, USA). Primers MCMV/CP-F and MCMV/CP-R were used to amplify the 887-bp fragment covering the CP region of MCMV. Primer sequences were listed in Table 3.

For potyvirus identification, a set of universal primers for potyviruses was used for RT-PCR as previously reported^34^. Briefly, first cDNA was synthesized using primer M4T [GTT TTC CCA GTC ACG AC -(T)_15_]. A degenerate primer Sprimer (GGN AAY AAY AGY GGN CAR CC, N = A, G, C or T; Y = T or C; R = A or G) and primer M4 (GTT TTC CCA GTC ACG AC) were used to amplify the 3′ terminal genome region of the potyvirus. The primers mentioned were kindly provided by Jianping Chen in Zhejiang Academy of Agricultural Sciences, Hangzhou, China.

### Sequence analysis

The sequences of four MCMV full-length cDNAs were determined by Sanger sequencing (Invitrogen, Shanghai, China), and the full-length sequences were assembled in SeqMan software (DNASTAR, Madison, WI, USA). The resulting sequences were aligned against all the available full-length MCMV sequences in the GenBank database using the software Mega5^35^. A phylogenic tree was then constructed with the neighbor-joining method provided in Mega5.

### Construction of infectious MCMV clone

Isolate MCMV-YN2 was randomly selected to construct an infectious clone. MCMV-YN2 cDNA was inserted behind a duplicated 35S promoter in the pCB301 vector. The binary vector pCB301^36^ harboring a duplicated 35S promoter (2 × 35S) and a hepatitis delta virus ribozyme (HDV_Rz) and a NOS terminator was kindly provided by Professor Xiaorong Tao (Nanjing Agriculture University, Nanjing, China). A full-length PCR product representing MCMV-YN2 was further amplified and digested with XhoI restriction enzyme. The 5′ fragment of the digested PCR product (MCMV nucleotides 1 to 3579) was ligated into the pCB301 vector pre-digested with StuI and SalI enzymes to generate pCB301-SX. The full-length PCR product was digested again with NheI enzyme, and the 3′ fragment product (MCMV nucleotides 748 to 4436) was ligated into the pCB301-SX vector at the NheI and SmaI site to generate pMCMV. This final clone contained a full-length MCMV-YN2 cDNA between the StuI and SmaI sites (Fig. 1). Primer sequences were listed in Table 3.

### Inoculation

Isolate MCMV-YN1 was used as a control (wild-type virus, wt). MCMV-YN1-infected maize leaves were ground in 0.1 M phosphate buffer (pH 7.0), and the sap was rubbed onto leaves of maize plants with 5–6 leaves^37^.

pMCMV described above were used to transform *Agrobacterium tumefaciens* GV3101 via electroporation. The transformants were then cultured overnight at 28 °C in YEP broth supplemented with 25 mg/L rifampicin, 50 mg/L kanamycin and 25 mg/L gentamicin. The *Agrobacterium* cells were pelleted by 10 min centrifugation at 6000× *g* and then re-suspended in an infiltration buffer (10 mM MES, pH 5.6, 10 mM MgCl_2_, and 200 μM acetosyringone) until the OD_600_ reached 1.0^38^. Maize seedlings of cultivars B73, Zhengdan958, Suyu No. 1, Huatian-Waxy Corn 072, Urban Beauty, Nongda108 and Zhe-Waxy Corn No. 5 were inoculated with *Agrobacterium* cells transformed with pMCMV. A 1-mL syringe was then used to inject maize seedlings 3 mm above the coleoptilar node with 0.5 ml of *Agrobacterium* cells harboring pMCMV. This injection site was previously reported to contain meristematic tissue and is susceptible to *Agrobacterium* invasion^39^.

### Northern blot assay and quantitative RT–PCR

Total RNA (10 μg) extracted from maize leaf tissue was electrophoresed in 0.8% (w/v) agarose formaldehyde gels and transferred to the Hybond N+ membranes (GE Amersham, PA, USA) by capillary transfer. RNA agarose gels stained with ethidium bromide were imaged under a UV light to estimate the loadings for each sample. The membranes were hybridized with a ^32^P-dCTP labeled MCMV-specific probe prepared with the random priming method (Prime-a-Gene Labeling System, Promega) as instructed by the manufacturer.

Quantitative RT-PCR (qRT-PCR) for the *Pyruvate orthophosphate dikinase* gene (PPDK) was performed using primers PPDK/F and PPDK/R. The *Elongation factor 1-alpha* (EF1α) gene was used as the internal controls. All qRT-PCR experiments were done in triplicate using three independent samples as described previously^40^.

### Western blot

Approximately 100 mg tissue from each maize leaf sample was homogenized in 0.2 ml protein extraction buffer (50 mM Tris-HCl, pH 6.8, 9 M urea, 4.5% sodium dodecyl sulfate [SDS] and 7.5% β-mercaptoethanol). The crude extracts were centrifuged at 12,000× *g* for 15 min at room temperature, and the resulting supernatant (15 μl per sample plus 15 μl 2× SDS loading buffer) was electrophoresed in 12.5% SDS-PAGE gels followed by transferring proteins to nitrocellulose membranes performed as previously reported^41^. The membranes were then probed with the MCMV-specific monoclonal antibody^31^ followed by a goat anti-mouse secondary antibody conjugated to horseradish peroxidase (Bio-Rad, Los Angeles, CA, USA).

### Electron microscopy

Symptomatic leaves were collected from the MCMV-infected plants and ground in water. The leaf extracts were loaded onto formvar-coated copper grids. After negative staining with 2% phosphotungstic acid, pH 6.7, the grids were examined for MCMV particles with a transmission electron microscope (TEM; H-7650, Hitachi, Japan) at 80 kV accelerating voltage.

For cytopathological studies, leaves were harvested from maize plants infected with MCMV or coinfected with MCMV and SCMV, then cut into small pieces (about 1 × 3 mm). The tissues were fixed in 2.5% glutaraldehyde and 1% osmium tetroxide in 100 mM phosphate buffer, pH 7.0, as described previously^42^. The fixed tissues were dehydrated through an ethanol series, then embedded in Epon 812 resin as instructed by the manufacturer (SPI-EM, Division of Structure Probe, West Chester, PA, USA). Ultrathin sections were cut and placed onto formvar-coated grids and stained with 2% uranyl acetate for 10 min followed by 2.5% lead citrate solution for 10 min. The stained sections were examined using the TEM as described.

## Additional Information

**How to cite this article**: Wang, Q. *et al*. Further characterization of *Maize chlorotic mottle virus* and its synergistic interaction with *Sugarcane mosaic virus* in maize. *Sci. Rep.* **7**, 39960; doi: 10.1038/srep39960 (2017).

**Publisher's note:** Springer Nature remains neutral with regard to jurisdictional claims in published maps and institutional affiliations.

## Figures and Tables

**Table 1:** Survey samples from Chinese provinces and ELISA results for MCMV and SCMV detection.

Province	Year	Total samples	MCMV only	MCMV + SCMV	SCMV only
Yunnan	2009	5	0	2	1
	2010	8	0	6	1
	2011	10	0	4	1
	2012	7	0	2	0
	2013	6	0	4	1
Sichuan	2011	10	0	4	1
	2012	18	0	6	0
	2013	10	0	5	2
Guangxi	2012	8	0	0	0
Guizhou	2012	10	0	0	1
Shandong	2012	15	0	0	2
Liaoning	2012	8	0	0	0
Heilongjiang	2012	12	0	0	0
He’nan	2012	11	0	0	2
Hebei	2012	12	0	0	3
Zhejiang	2012	21	0	0	5

**Table 2:** Infectivity of the infectious clone pMCMV.

*Agrobacterium*	Inoculation experiments	Inoculated plants	Infected plants*	Infectivity (%)
GV3101 (pMCMV)	1	30	20	66.7
	2	30	23	76.6
	3	30	22	73.3
GV3101 (pCB301)	1	15	0	0
	2	15	0	0
	3	15	0	0

^*^The infected plants were counted at 20 dpi.

**Table 3:** Primers and sequences used for PCR.

Primers	Primer sequences, 5′ → 3′	Purpose
M1F	AGGTAATCTGCGGCAACAGACCCCAACG	For clone of MCMV cDNA
M2R	GGGCCGGAAGAGAGGGGCATTAC	
MCMV/CP-F	ACAGGACACCGTTGCCGTTTAT	For MCMV detection
MCMV/CP-R	CGATTTAGGCTCCCAGACACTT	
SCMV/F	GTGTGGAATGGTTCACTCAAAGCTG	For SCMV detection
SCMV/R	GGTGTTGCAATTGGTGTGTACACG	
PPDK/F	CGCCGATACAGACGACCAAAAAGAGG	For amplification of *PPDK*
PPDK/R	CCCAGCAGTTCCTTCATGGTCTTGT	
EF1α/F	TTCACACTTGGTGTGAAGCAGATG	For amplification of *EF1α*
EF1α/R	TTGTATCCAACCTTCTTCAGGTAGG	
